# Familial risk in testicular cancer as a clue to a heritable and environmental aetiology

**DOI:** 10.1038/sj.bjc.6601714

**Published:** 2004-03-16

**Authors:** K Hemminki, X Li

**Affiliations:** 1 Department of Biosciences at Novum, Karolinska Institute, 141 57 Huddinge, Sweden; 2 Division of Molecular Genetic Epidemiology, German Cancer Research Center (DKFZ), Im Neuenheimer Feld 580, 69120 Heidelberg, Germany

**Keywords:** testicular cancer, seminoma, teratoma, familial risk, genetics, hereditary

## Abstract

We used the nation-wide Swedish Family-Cancer Database to examine the risk for testicular cancer in offspring through parental and sibling probands. Among 0–68-year-old offspring, 4082 patients had testicular cancer in years 1961–2000, among whom 68 (1.67%) had an affected father/brother. Standardized incidence ratios (SIRs) for familial risk were four-fold when a father and nine-fold when a brother had testicular cancer. Histology-specific risks (for the testicular cancer) were similar for sons of affected fathers, but were higher among brothers for teratoma and seminoma than for mixed histologies. Standardized incidence ratios for either histology depended on the age difference between the brothers: 10.81 when the age difference was less than 5 years compared to 6.69 for a larger age difference. Parental colorectal, pancreatic, lung and breast cancer and non-Hodgkin's lymphoma and Hodgkin's disease were associated with seminoma among sons. Seminoma risk was also increased when a sibling had melanoma. Teratoma was associated with parental lung cancer and melanoma. The high familial risk may be the product of shared childhood environment and heritable causes. Familial cases of fraternal pairs with an early-onset teratoma represent a challenge for gene identification.

The incidence of testicular cancer has increased in many countries, including Sweden, two- to four-fold over the last half a century, for unknown reasons (Bergstrom *et al*, 1996; Centre for Epidemiology, 2002). Of the two main types, seminomas and nonseminomas, the latter include teratomas with somatic differentiation and undifferentiated embryonal tumours (Kumar *et al*, 1997). However, some 60% of germ cell tumours of the testis contain multiple histological types and only 40% contain a single histological type (Kumar *et al*, 1997). The aetiology of testicular cancer remains largely unknown; some identified or suggested risk factors include undescended testis (cryptorchidism), a prior history of cancer in one testis (the opposite testis is at increased risk), *in utero* hormonal exposures, perinatal factors and family history of testicular cancer (Forman *et al*, 1992,^1994^; Heimdal *et al*, 1997; Moller and Skakkebaek, 1997; Swerdlow *et al*, 1997; Westergaard *et al*, 1998; Jacobsen *et al*, 2000; Hemminki and Mutanen, 2001). Compared to the relatively high incidence of testicular cancer in Sweden, first-generation immigrants generally show a decreased risk of testicular cancer compared to the natives, but this difference disappears in the next generation; this effect is marked among the sons of Finnish immigrants, whose risk is doubled compared to their fathers (Hemminki and Li, 2002b; Hemminki *et al*, 2002). Among the sons of the Danish immigrants, an equally large but opposite change takes place (Hemminki and Li, 2002a).

In the present study, we have examined the genetic epidemiology of histology-specific testicular cancer in order to distinguish the contribution of heritable and environmental effects to etiology. Compared to the previous testicular cancer study from the Swedish Family-Cancer Database, an extended population and hence some 50% more familial testicular cancer cases are available (Dong *et al*, 2001), allowing analysis by age of onset through parental and fraternal probands. This, the largest cohort study of familial testicular cancer, offers new insight into the aetiology of the disease.

## SUBJECTS AND METHODS

Statistics Sweden maintains a ‘Multigeneration Register’ in which offspring, born in Sweden in 1932 and later, are registered with their parents (as declared at birth) and they are organized as families (Hemminki *et al*, 2001a). Information on the Database is available at the Nature Genetics website as ‘Supplementary information’ (Hemminki and Granstrom, 2002). The data on families and cancers have a complete coverage, barring some groups of deceased offspring born in the 1930s and who died before 1991. Although this small group of offspring with missing links to parents has a negligible effect on the estimates of familial risk (Hemminki and Li, 2003), we limited the present study to offspring whose parents were known, to eliminate this possible source of bias. The ‘Multigeneration Register’ was linked using the individually unique national registration number to the Cancer Registry for the years 1958–2000. Cancer registration is considered now to be close to 100% complete (Centre for Epidemiology, 2002).

The registered site of cancer is as a four-digit diagnostic code based on the 7th revision of the International Classification of Diseases (ICD-7). The following ICD-7 codes were grouped: ‘upper aerodigestive tract’ cancer codes 161 (larynx) and 140–148 (lip, mouth, pharynx), except for code 142 (salivary glands), ‘lymphoma’ codes 200, 202 (non-Hodgkin lymphoma), 201 (Hodgkin's disease) and ‘leukemia’ codes 204–207 (leukemias), 208 (polycytemia vera) and 209 (myelofibrosis). Rectal cancer, ICD-7 code 154, was subdivided into the anus (squamous cell carcinoma, 154.1) and mucosal rectum (154.0). Basal cell carcinoma of the skin is not registered in the Cancer Registry. Up to 1992, the histology of testicular cancers as in the Cancer Registry (WHO/HS/CANC/24.1 Histology Code) was used, to define seminoma (pathology codes 066) and teratoma (826, also including embryonal tumours). From 1993, ICD-O-2/ICD with histopathological data according to the Systematized Nomenclature of Medicine (SNOMED, http://snomed.org) was used, referred to here as ‘SNOMED’.

Standardized incidence ratios (SIRs) were used to measure cancer risks for sons (i.e., offspring) according to the occurrence of cancers in their families. When more than two affected sons were found in any family, they were counted as independent events. Standardized incidence ratios were calculated for sons whose parents or brothers had the same, concordant cancer, that is, using parents or brothers as probands. Follow-up started for each offspring at birth, immigration or January 1, 1961, whichever came latest, and terminated on diagnosis of the first cancer, death, emigration, or the closing date of the study, December 31, 2000.

Parents’ ages were not limited but sons were 0–68 years of age. All tumour incidence rates were based on the data in the Family-Cancer Database, and they were essentially similar to rates in the Swedish Cancer Registry. Rates were standardized to the European population. Standardized incidence ratios were calculated as the ratio of observed (O) to expected (E) number of cases. The expected numbers were calculated from 5-year-age-, sex-, tumour type-, period- (5-year bands), socioeconomic status- (six groups) and residential area- (three groups) specific standard incidence rates for all sons lacking a family history (Esteve *et al*, 1994). Confidence intervals (95% CI) were calculated assuming a Poisson distribution (Esteve *et al*, 1994). Risks for siblings were calculated using the cohort method, described elsewhere (Hemminki *et al*, 2001b).

The kappa statistic was used as the measure of agreement between histologies: (observed number of cases−expected number of cases)/(1−expected number of cases) (Armitage and Berry, 1994). The kappa can assume values between −1 and 1; 0 shows a complete chance occurrence and −1 or 1 show a completely determined occurrence. Values between 0.40 and 0.60 are considered moderately determined occurrences. A negative kappa value would, in the present context, indicate a determined occurrence of a discordant histology, which is biologically unlikely, so we present only positive values of kappa in this paper.

## RESULTS

The Family-Cancer Database, which covered years 1961–2000 from the Swedish Cancer Registry, included 4082 testicular cancers in sons of ages 0–68 years and 3878 fathers with testicular cancer (Table 1

**Table 1:** Numbers of cases of testicular cancer in sons and fathers

	**Son**				**Father**			
**Histopathological type**	**No.**	**%**	**Mean age**	**Median age**	**No.**	**%**	**Mean age**	**Median age**
*Pathology (1961–2000)*								
Seminoma	2032	49.8	35.8	35	2293	59.1	41.1	39
Teratoma	1974	48.4	27.5	27	1483	38.2	33.1	33
Others	76	1.9	29.9	27	102	2.6	46.7	43
All	4082	100.0	31.7	31	3878	100	38.2	36
								
*SNOMED (1993–2000)*								
Seminoma	871	56.4	37.7	36	673	66.2	42.0	39
Teratoma	385	25.0	28.8	27	193	19.0	35.6	33
Embryonal carcinoma	228	14.8	28.4	27	111	10.9	34.7	32
Others	59	3.8	28.5	28	40	3.9	42.6	37
All	1543	100.0	33.8	33	1017	100.0	40.0	37

). Seminoma accounted for 49.8% and teratoma 48.4% in sons, while in fathers the proportions were 59.1 and 38.2%, respectively. Seminoma showed a 6–8-year later median age of onset than teratoma (35 *vs* 27 in offspring and 39–33 in fathers). According to the SNOMED histology, covering years 1993–2000, embryonal carcinoma accounted for 14.8% in sons and 10.9% in fathers. The age-specific incidence rates of testicular cancer among sons are shown in Figure 1

**Figure 1:**
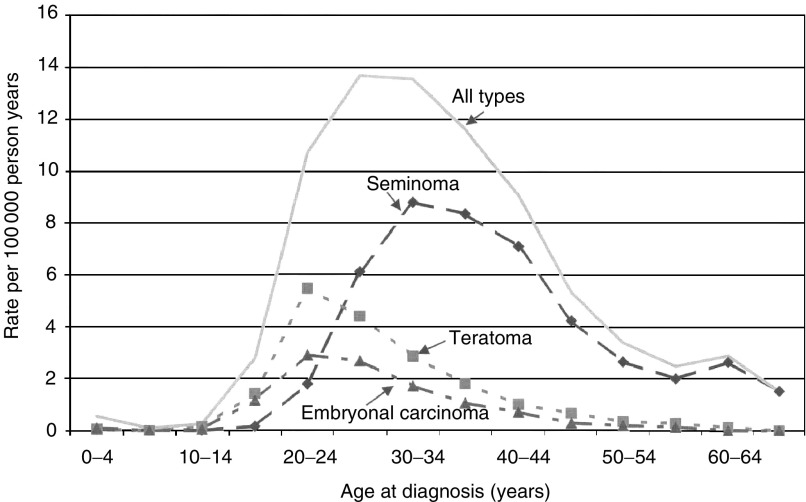
Age-specific incidence of testicular cancer in offspring according to the SNOMED histology in years 1993–2000.

according to the SNOMED histology. The peak incidence for teratoma and embryonal carcinoma occurred at ages 20–24 years, and for seminoma showed a peak incidence at 30–34 years.

Table 2

**Table 2:** SIR for histological type of testicular cancer in brothers by age difference

		**Brothers ages <5 years**				**Brothers ages ⩾5years**			
**Histological types**	**Age at diagnosis**	**O**	**SIR**	**95% CI**		**O**	**SIR**	**95% CI**	
Seminoma	0–24	1	11.49	0.00	65.89	0			
	>24	14	**10.62**	**5.79**	**17.87**	11	**6.81**	**3.38**	**12.22**
	All	15	**10.67**	**5.96**	**17.65**	11	**6.40**	**3.18**	**11.49**
									
Teratoma	0–24	5	**11.61**	**3.66**	**27.31**	5	**10.74**	**3.39**	**25.26**
	>24	9	**10.18**	**4.61**	**19.41**	6	**5.73**	**2.06**	**12.56**
	All	14	**10.65**	**5.80**	**17.91**	11	**7.27**	**3.61**	**13.06**
									
All types	0–24	7	**13.08**	**5.18**	**27.10**	5	**8.54**	**2.69**	**20.08**
	>24	23	**10.27**	**6.50**	**15.44**	17	**6.29**	**3.66**	**10.09**
	All	30	**10.81**	**7.29**	**15.45**	22	**6.69**	**4.19**	**10.15**

Bold type: 95% CI does not include 1.00. O=observed; SIR=standardised incidence ratio; CI=confidence interval.

presents risks for the histological types among brothers according to their age difference. Those born less than 5 years apart had higher risks, particularly at ages over 24 years, but below age 25 for the teratoma showed no age difference.

Age- and histology-specific familial risk for testicular cancer was analysed using fathers or brothers as probands (Table 3

**Table 3:** SIR for histological types of testicular cancer in the offspring of paternal and fraternal probands

		**Seminoma**				**Teratoma**				**All types**			
**Histological types in proband**	**Age at diagnosis**	**O**	**SIR**	**95% CI**		**O**	**SIR**	**95% CI**	**O**	**SIR**	**95% CI**		
*Paternal proband*													
Seminoma	0–24	0				3	4.62	0.87	13.69	3	3.78	0.71	11.18
	>24	4	3.52	0.92	9.11	3	3.6	0.68	10.66	7	**3.50**	**1.39**	**7.26**
	All	4	3.18	0.83	8.23	6	**4.05**	**1.46**	**8.87**	10	**3.58**	**1.71**	**6.61**
													
Teratoma	0–24	0				3	**8.64**	**1.63**	**25.58**	3	**7.2**	**1.36**	**21.32**
	>24	2	4.01	0.38	14.76	0				2	2.28	0.22	8.39
	All	2	3.61	0.34	13.27	3	4.2	0.79	12.45	5	**3.87**	**1.22**	**9.1**
													
All types	0–24	0				7	**6.85**	**2.71**	**14.18**	7	**5.63**	**2.23**	**11.66**
	>24	6	**3.53**	**1.27**	**7.73**	3	2.42	0.46	7.15	9	**3.02**	**1.37**	**5.75**
	All	6	**3.19**	**1.15**	**6.98**	10	**4.42**	**2.1**	**8.15**	16	**3.78**	**2.16**	**6.16**
													
*Fraternal proband*													
Seminoma	0–24	1	11.7	0	67.04	3	**7.79**	**1.47**	**23.07**	**5**	**10.29**	**3.25**	**24.22**
	>24	15	**9.65**	**5.38**	**15.95**	6	**6.11**	**2.2**	**13.38**	21	**8.15**	**5.03**	**12.47**
	All	16	**9.75**	**5.56**	**15.88**	9	**6.58**	**2.98**	**12.55**	26	**8.49**	**5.54**	**12.45**
													
Teratoma	0–24	0				7	**14.35**	**5.69**	**29.74**	7	**11.53**	**4.57**	**23.89**
	>24	9	**6.88**	**3.12**	**13.12**	9	**9.95**	**4.51**	**18.98**	18	**8.02**	**4.74**	**12.7**
	All	9	**6.39**	**2.9**	**12.18**	16	**11.50**	**6.55**	**18.71**	25	**8.76**	**5.67**	**12.96**
													
All types	0–24	1	5.27	0	30.2	10	**11.16**	**5.31**	**20.6**	12	**10.71**	**5.51**	**18.76**
	>24	25	**8.52**	**5.51**	**12.59**	15	**7.77**	**4.33**	**12.84**	40	**8.1**	**5.78**	**11.03**
	All	26	**8.32**	**5.43**	**12.21**	25	**8.84**	**5.72**	**13.07**	52	**8.58**	**6.41**	**11.26**

Bold type: 95%CI does not include 1.00. O=observed, SIR=standardised incidence ratio; CI=confidence interval.

). The overall SIRs were approximately two-fold higher between brothers (8.58) than between sons and fathers (3.78). The risks were slightly higher for unmixed histologies compared to the mixed histologies for all significant SIRs. Among brothers, the SIR for concordant teratoma was 11.50, compared to 9.75 for seminoma. Concordant teratoma showed the highest SIR (14.35) among brothers aged 0–24 years. The proportion of sons with testicular cancer who had an affected father or brother was 1.67% (68 familial cases from Table 3 to 4082 cases from Table 1). Median ages of the familial cases did not differ from those of all cases (data not shown).

**Figure 2:**
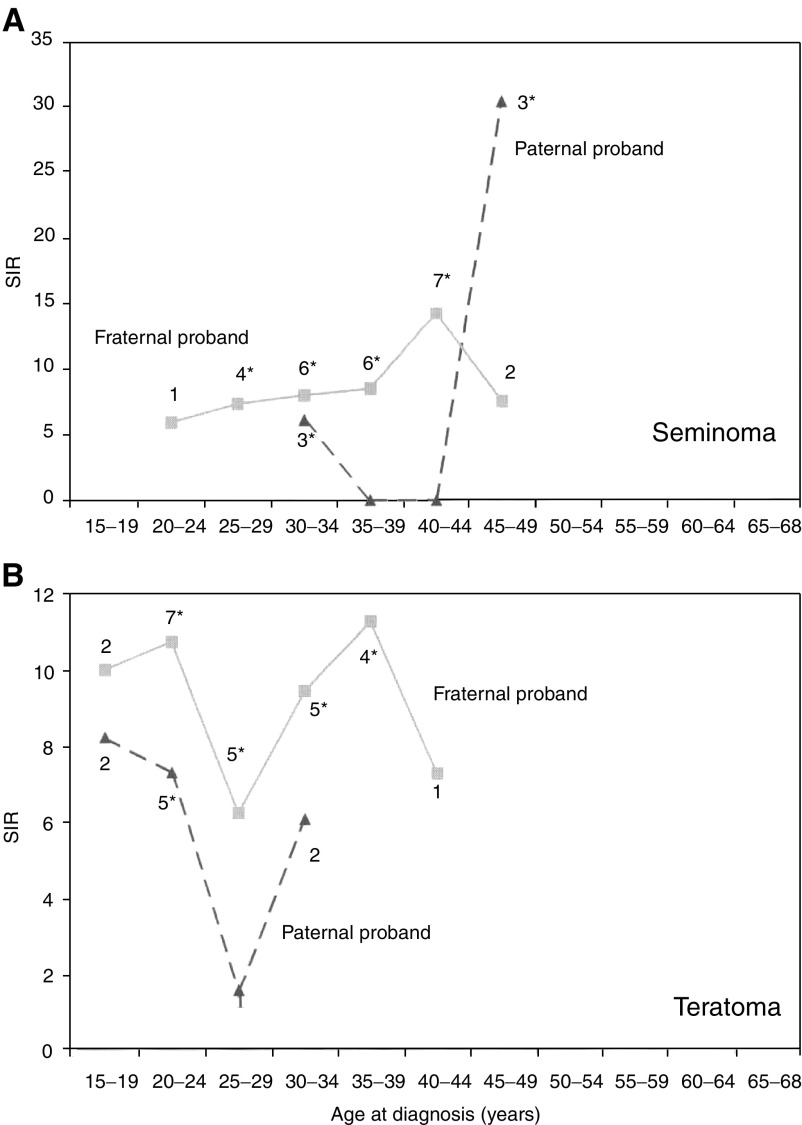
Age-specific SIR for histological type of testicular cancer in the offspring of paternal and fraternal probands: (**A**) seminoma; (**B**) teratoma. The numbers of cases are shown for each age group. ^*^ Shows that the 95% CI for the SIR did not include 1.00.

Age-specific familial SIR for seminoma is shown in Figure 2A

for sons of fathers and among brothers with testicular cancer; Figure 2B shows the curves for teratoma. The shapes of the curves for teratoma resemble each other independent of proband status, with two peaks at 15–24 and 30–39 years. For seminoma, the peak SIR was observed at ages 40–44 years among brothers, and at 45–49 years among sons of affected fathers.

We analysed using SNOMED histopathology, available only from 1993 (data not shown), that teratoma showed an age peak at 15–29 years in sons of fathers with testicular cancer (*N*=3, SIR=8.48, 95% CI 1.60–25.10). Among brothers, both SNOMED types showed peak SIRs at 30–44 years (seminoma, *N*=10, SIR=11.74, 95% CI 5.59–21.68; teratoma, *N*=5, SIR=26.16, 95% CI 6.26–61.55). No familial cases were found for embryonal carcinoma.

Table 4

**Table 4:** SIR for testicular cancer in sons of parents and among siblings with cancer

	**Paternal**								**Fraternal**							
	**Seminoma**				**Teratoma**				**Seminoma**				**Teratoma**			
**Cancer sites**	**O**	**SIR**	**91% CI**		**O**	**SIR**	**95% CI**		**O**	**SIR**	**95% CI**		**O**	**SIR**	**95% CI**	
Upper aerodigestive tract	15	0.83	0.46	1.37	17	1.19	0.69	1.19	2	0.84	0.08	3.11				
Oesophagus	12	**1.94**	**1.00**	**3.40**	5	1.18	0.34	2.55	2	4.19	0.39	15.41				
Stomach	34	0.99	0.68	1.38	18	0.77	0.45	1.21	1	0.59	0.00	3.36				
Colorectum^a^	123	**1.37**	**1.14**	**1.64**	80	1.19	0.95	1.49	5	0.73	0.23	1.72	4	0.94	0.24	2.43
Liver	21	0.93	0.57	1.42	13	0.80	0.42	1.36	1	0.73	0.00	4.20				
Pancreas	35	**1.52**	**1.06**	**2.12**	21	1.26	0.78	1.93	1	0.74	0.00	4.22	2	2.37	0.22	8.70
Lung	77	**1.32**	**1.04**	**1.65**	64	**1.40**	**1.08**	**1.78**	3	0.56	0.11	1.66	2	0.61	0.06	2.23
Breast	120	**1.28**	**1.06**	**1.53**	83	1.02	0.81	1.26	25	1.00	0.65	1.48	15	0.91	0.51	1.51
Cervix	14	0.79	0.43	1.33	17	1.14	0.66	1.83	5	0.99	0.31	2.33	1	0.26	0.00	1.50
Endometrium	24	1.12	0.72	1.67	16	0.95	0.54	1.54	4	1.30	0.34	3.36				
Uterus, other^b^	5	1.86	0.59	4.38	6	2.69	0.97	5.89								
Ovary	23	1.18	0.75	1.78	20	1.26	0.77	1.95	3	0.71	0.13	2.11				
Prostate	113	1.04	0.85	1.25	81	1.01	0.80	1.25	3	0.63	0.12	1.86	2	0.78	0.07	2.87
Testis	6	**3.27**	**1.18**	**7.17**	10	**4.50**	**2.15**	**8.32**	26	**8.46**	**5.52**	**12.41**	25	**8.95**	**5.79**	**13.23**
Kidney	23	0.89	0.56	1.33	12	0.60	0.31	1.06	4	1.42	0.37	3.67	2	1.07	0.10	3.94
Urinary bladder	34	0.90	0.63	1.26	34	1.18	0.82	1.66	1	0.27	0.00	1.56	3	1.32	0.25	3.91
Melanoma	29	1.32	0.88	1.89	39	**1.84**	**1.31**	**2.52**	17	**1.85**	**1.08**	**2.97**	4	0.58	0.15	1.51
Skin, squamous cell	28	1.11	0.74	1.61	15	0.82	0.46	1.36	3	1.34	0.25	3.98	3	2.05	0.39	6.07
Nervous system^c^	31	1.30	0.88	1.85	27	1.29	0.85	1.87	8	0.95	0.40	1.88	9	1.30	0.59	2.47
Thyroid gland	6	0.93	0.34	2.04	6	1.04	0.37	2.27	3	1.14	0.21	3.37	3	1.51	0.28	4.46
Endocrine glands	15	1.07	0.59	1.76	16	1.32	0.75	2.16	3	0.75	0.14	2.23	3	1.06	0.20	3.14
Connective tissue	5	0.99	0.31	2.32	4	0.94	0.24	2.43	3	2.26	0.43	6.70	2	1.86	0.18	6.84
Non-Hodgkin's lymphoma	35	**1.56**	**1.08**	**2.17**	22	1.19	0.75	1.81	3	0.65	0.12	1.92	6	1.85	0.66	4.04
Hodgkin's disease	9	**2.40**	**1.09**	**4.58**	0				2	1.03	0.10	3.79				
Myeloma	12	1.04	0.54	1.83	8	0.93	0.40	1.85								
Leukaemia	25	1.18	0.76	1.74	19	1.16	0.69	1.81	3	0.78	0.15	2.31	1	0.30	0.00	1.73
All	874	**1.19**	**1.11**	**1.27**	653	**1.13**	**1.04**	**1.22**	131	1.19	0.99	1.41	87	1.13	0.91	1.40

Bold type: 95% CI does not include 1.00. O=observed; SIR=standardised incidence ratio; CI=confidence interval.

a Three colorectal cancer-affected mothers had each two sons with testicular cancer, giving an SIR=7.49, 95% CI=1.41–22.18; of whom two pairs of bothers had seminoma, giving SIR=11.07 and 95% CI=1.04–40.71.

b For all types of testicular cancer by mother's uterine cancer, SIR=2.40, 95% CI=1.23–4.20.

c For all types of testicular cancer by father's nervous system cancer, SIR=1.50, 95% CI=1.03–2.10.

presents associations of testicular cancer with other cancers in families, including mothers and sisters. Both seminoma (1.19) and teratoma (1.13) were increased when parents had any cancer (only associations at discordant sites being considered). For seminoma, a significantly increased risk was found when parents had colorectal, pancreatic, lung and breast cancer and non-Hodgkin's lymphoma and Hodgkin's disease. Seminoma was also increased when a sibling (brother or sister) had melanoma. Teratoma was associated with parental lung cancer and melanoma. For testicular cancer as a whole, an increased risk was found when the mother was diagnosed with ‘other uterine tumours’ or the father with nervous system cancer; these uterine tumours included three leiomyosarcomas, two adenocarcinomas, two chorioncarcinomas, two embryonal sarcomas, one stroma cell sarcoma and two unspecified sarcomas. It was also noteworthy that there were three mothers with colorectal cancer, each with two sons with testicular cancer (SIR=7.49, 95% CI=1.41–22.18), of whom two pairs of brothers had seminoma (SIR=11.07, 95% CI=1.04–40.71).

The kappa test was applied to assess the histological concordance of testicular cancer (data not shown). The overall value was 0.01 between sons and fathers, and 0.26 among brothers; among brothers, the kappa value of seminoma was 0.23 and that of teratoma was 0.31.

## DISCUSSION

The Swedish Family-Cancer Database contains national family data linked to the Swedish Cancer Registry. The inability to link some 10% of deceased offspring diagnosed with cancer to their parents (see Subjects and Methods) may cause small errors in the familial risks of fatal cancers. However, testicular cancer has had a good prognosis during the past decades. The missing links predominantly influence those born in the 1930s and who died before 1991, and we have not observed a difference in familial risks in comparing different diagnostic periods (Hemminki and Li, 2003). We conclude that this gap in parental links has no large effect on the present estimates. The markedly improved survival in testicular cancer may also be a source of bias, but adjustment for period should have minimized this. The many comparisons are relevant and, undoubtedly, some associations were due to chance; consistency within this study and with other studies, as well as biological plausibility, need assessing for causal inference.

Familial occurrence of testicular cancer is well recognized but rare. The proportion of sons with testicular cancer who had an affected father or brother was 1.67% in the present study, consistent with previous studies, reporting affected first-degree relatives in 1.0–2.8% of cases (Heimdal *et al*, 1996; Westergaard *et al*, 1996; Dieckmann and Pichlmeier, 1997; Hemminki and Czene, 2002). Even the present overall familial risks of 3.78 (son–father) and 8.58 (brothers) were in line with the literature (Westergaard *et al*, 1996; Dieckmann and Pichlmeier, 1997; Heimdal *et al*, 1997; Dong *et al*, 2001). The SNOMED data, covering cases diagnosed between 1993 and 2000, showed an even larger difference between the two proband groups, 3.12 (son–father) and 9.62 (brothers), respectively. There was no large difference in familial risks between seminomas and teratomas among father–son pairs. However, there appeared to be a large difference in the familial risk between the pure and mixed histological types among brothers. The risks ranged from 12 (teratoma–teratoma) and 10 (seminoma–seminoma) for pure histologies to six for mixed histologies. Consistent with these findings, the kappa test was 0.01 between sons and fathers, whereas between brothers seminoma showed a value of 0.23 and teratoma a value of 0.31. These are still low values, but the interpretation is difficult because of the relatively small number of cases. The lower kappa values between sons and fathers than between brothers may be due to a more defined disease phenotype within one generation than between two generations.

The higher familial risk for testicular cancer among brothers than father–son pairs may suggest the involvement of a recessive mode of inheritance or an X-linked susceptibility locus in the aetiology of testicular cancer, consistent with the segregation analysis and the gene-mapping findings (Heimdal *et al*, 1997; Rapley *et al*, 2000). Such results would point to the importance of the maternal lineage of inheritance and perhaps also maternally exerted environmental factors. The difference in SIR among brothers close in age (10.81) compared to those further apart (6.69) suggests environmental effects. Testicular cancer has been reported as the site with the highest proportion of childhood-shared environmental effects in a family study of all major cancers (Czene *et al*, 2002). Although both histological types showed the effect of age difference, the risks for early-onset teratoma appeared to be least influenced by the age difference. These results suggest that environmental factors during childhood and adolescence influence the risk of contracting a late-onset testicular cancer (Hemminki *et al*, 2002; Hemminki and Li, 2002b). Identifying these factors might explain the riddle of increasing incidence trends and also the difference between immigrants and their sons (Hemminki and Li, 2002a). On the other hand, the search for heritable effects should target brother pairs with an early-onset teratoma.

Age-specific familial risks of Figure 2 showed the highest risks for seminoma in the 40 s, for both brothers and son–father pairs. For teratoma, two discrete peaks were noted, particularly among brothers. The early-onset teratoma peak (20–24 years) coincided with its peak incidence, but the late-onset peak (35–39 years) occurred 15 years later and close to the peak fraternal risk of seminoma. Based on the risks by age difference, the younger teratoma component may be the most heritable familial component, whereas the later component of teratoma and seminoma may have a strong environmental origin.

In families of seminoma patients, associations were found with colorectal, pancreatic, lung and breast cancer and non-Hodgkin's lymphoma and Hodgkin's disease among parents. Among brothers, there was an association with seminoma and melanoma. Teratoma was associated with parental lung cancer and melanoma. However, no association has been found for primary melanoma or lung cancer following first testicular cancer (Dong *et al*, 2001). Testicular cancer was associated with mothers’ unusual uterine tumours, including chorionepithelioma (SIR=2.40, 95% CI 1.23–4.20). No oestrogen-related cancer risks were observed in mothers of testicular cancer patients in a Danish study (Kroman *et al*, 1996). In the 26 families in our study with two sons with testicular cancer, three had mothers with colorectal cancer (SIR=7.49, 95% CI=1.41–22.18), of whom two pairs of brothers had seminoma (SIR=11.07, 95% CI=1.04–40.71), but none had a father with testicular cancer. However, there is no previous evidence of an association between testicular and colorectal cancers, but multiple testing may have resulted in associations due to chance.

In summary, the present study may offer some explanation to the inability in finding susceptibility genes for testicular cancer. The high familial risk may be the product of shared childhood environment and heritable causes and so may be difficult to untangle. Identifying any relevant environmental factors will be challenging but may explain some of the changes in testicular cancer incidence. For gene identification, fraternal pairs with teratoma below age 25 may be particularly useful.
